# Beyond Basic Diversity Estimates—Analytical Tools for Mechanistic Interpretations of Amplicon Sequencing Data

**DOI:** 10.3390/microorganisms10101961

**Published:** 2022-10-01

**Authors:** Anna Trego, Ciara Keating, Corine Nzeteu, Alison Graham, Vincent O’Flaherty, Umer Zeeshan Ijaz

**Affiliations:** 1 Microbial Ecology Laboratory, School of Biological and Chemical Sciences and the Ryan Institute, University of Galway, University Road, H91 TK33 Galway, Ireland; 2 Institute of Biodiversity, Animal Health & Comparative Medicine, The University of Glasgow, Oakfield Avenue, Glasgow G12 8LT, UK; 3 Water Engineering Group, School of Engineering, The University of Glasgow, Oakfield Avenue, Glasgow G12 8LT, UK

**Keywords:** 16S rRNA, amplicons, ecology, microbiome, sequence analysis

## Abstract

Understanding microbial ecology through amplifying short read regions, typically 16S rRNA for prokaryotic species or 18S rRNA for eukaryotic species, remains a popular, economical choice. These methods provide relative abundances of key microbial taxa, which, depending on the experimental design, can be used to infer mechanistic ecological underpinnings. In this review, we discuss recent advancements in in situ analytical tools that have the power to elucidate ecological phenomena, unveil the metabolic potential of microbial communities, identify complex multidimensional interactions between species, and compare stability and complexity under different conditions. Additionally, we highlight methods that incorporate various modalities and additional information, which in combination with abundance data, can help us understand how microbial communities respond to change in a typical ecosystem. Whilst the field of microbial informatics continues to progress substantially, our emphasis is on popular methods that are applicable to a broad range of study designs. The application of these methods can increase our mechanistic understanding of the ongoing dynamics of complex microbial communities.

## 1. High-Throughput Sequencing: Widely Used, but Under-Explored

The past several decades have seen strategic advancements in sequencing technologies [1,2], which have shaped our fundamental understanding of the human genome [3], marine and terrestrial biogeochemical cycling [4,5], and global biodiversity [6]. This, in turn, has had significant impacts in practice for clinical medicine, forensics, environmental engineering and biotechnology. For example, sequencing has enabled achievements such as the study of the ‘unculturable majority’—all the organisms that cannot be successfully cultured in the lab [6,7]. This has improved our understanding of biodiversity, ecology and evolution and identified previously unknown organisms which have revolutionized biotechnology and medicine [8]. Furthermore, the application of sequencing technologies to cancer research has facilitated the identification of disease-specific drivers, mutational signatures, tumor mutational burden and neo-antigens, offering the promise of personalized patient care [9]. Moreover, the recent (2020) complete, telomere-to-telomere sequencing of the human genome [10] surely signifies that in terms of sequencing, the best is yet to come. 

Such advancements are often the result of cutting-edge sequencing approaches such as whole genome sequencing (WGS) [11,12], sequencing of transcriptomes (RNA-seq) [13,14], long-read sequencing (Oxford NanoPore, MinION, SMRT-seq) [15], or single cell sequencing (scRNA-seq) [16]. For microbial ecologists, shotgun metagenomics (the untargeted WGS of all the microbial genomes present in a sample) has enabled large-scale investigations into complex microbiomes [12]. It is a powerful tool which can provide taxonomic profiles, recover novel genomes and investigate the functional potential of a community. While the past 15 years have resulted in substantial reductions in terms of sequencing costs, the downstream data processing of metagenomes remains a significant challenge, particularly in view of co-assembly of sequence reads from all samples—a step required to bin the contigs into metagenomic assembled genomes (MAGs) later on. The co-assembly process typically requires holding debruijn graphs in memory, and with the enormous amount of sequencing data generated from the latest sequencing technologies, such as NovaSeq from Illumina, the memory footprint (RAM) often becomes a bottleneck. In addition, to capture accurate diversity, sequencing depth becomes an important factor, particularly for rarer genomes. Therefore, in terms of economics and computational requirements, WGS is often used on a small subset of samples, typically informed by a larger short-read amplicons dataset. 

The choice between WGS and short-read amplicons also comes down to the aim of the proposed study, whether the goal is to explore microbial ecology [17,18,19], or to discover novel genomes [20]. Not only is sequencing short-read amplicons cheaper [21], but the downstream analysis is more accessible, faster and can access ever-improving reference databases [22,23,24,25]. Combined, this results in a more economical way to test hypotheses. Indeed, the widespread application of amplicon sequencing has resulted in a wealth of taxonomic and phylogenetic information about a variety of complex microbiomes [26,27]. It should, however, be noted that amplicon sequencing has its own limitations including short read lengths, sequencing errors, and relative abundances that will always be biased towards certain species due to primer-based amplification and different numbers of the 16S gene [28].

Whilst there is a preponderance of amplicon-based studies, published across a diverse range of fields, more often than not, they include only basic analyses, including: (i) diversity estimates (within samples and between samples); and (ii) how microbial species differ or remain persistent in either a case–control or spatial/temporal gradient. These analyses may serve the basic ambitions of a given study, however, to gain a mechanistic understanding with ecological relevance, we need to go beyond basic analyses. 

Almost daily, novel methods and models are being reported for analyzing amplicon sequencing data [29,30,31,32,33]. Often, such methods are applied in the context of the human gut microbiome, but they are easily adaptable for other fields within microbial ecology—as long as the study design and datasets are suitable. These new analytical tools generally fall into one (or two) of several categories: methods which (i) quantify microbial community assembly; (ii) map network inferences; (iii) monitor spatial/temporal dynamics; (iv) integrate various types of datasets (integrative ‘omics); (v) identify discriminant or differential taxa; (vi) find correlations between species abundance and environmental variables; or (vii) predict functional patterns. Given that high-throughput sequencing is still being frequently employed, but that new analytical tools are slow to be utilized, our aim in this review is two-fold: (i) to highlight trends in experimental design and data processing for studies utilizing short-read amplicon sequencing; and (ii) to highlight the utility of more sophisticated sequence analyses, showcasing several easily applied, new analytical techniques for enhanced mechanistic understanding of complex microbiomes.

## 2. Study Design Considerations: Planning for Statistics

The type of downstream microbiome analysis that can be applied is entirely dependent on the original hypotheses being tested and study design parameters. Therefore, it is always wise to plan ahead for the type of analysis that will generate meaningful data and best test the experimental hypotheses. Microbiome analysis is hugely affected by a lack of harmonized protocols, including sample collection methods, storage, processing and downstream analysis [12,34,35]. Furthermore, variation in sample handling, sampling size, controls [36], the choice of extraction method [37], sequencing blank/mock communities, choice of sequencing platform and the downstream analysis [38,39] can contribute to the introduction of various biases leading to inconsistent and incomparable results. Therefore, in microbiome research, all these parameters must be carefully chosen and defined [34,35]. Whichever approach we use, we want it to be minimally biased against error.

At the bare minimum, microbial community samples are obtained in a case–control relationship (changing physico-chemical parameters in environmental datasets, or, pathology versus healthy controls in medical datasets). In some cases, the sampling is done over spatial or temporal gradients, particularly in intervention studies where the goal is to modulate the microbiome through some sort of treatment. This becomes convoluted for human microbiome datasets where there is an additional complication of ‘pairedness’ by virtue of multiple samples collected from the same subject over the course of the treatment [40]. Moreover, in other cases, there is a multifactorial or nested design [41]. For all these different study designs, there is no unified statistical framework, and each type will require a different set of statistical tools. 

Finally, an important consideration for any microbiome study is the number of replicate samples sequenced per experimental condition. Statistically, the required number of samples depends on the effect size—the magnitude of difference between categories [36]. Tools such as “Evident” [42] have been developed to help estimate sample size based on projected effect size and records from similar studies [36]. In addition to thinking about the statistical power of the number of replicates, various types of downstream statistical models have their own requirements. While the number of replicates required per category is under constant debate, from our experience, replicate numbers can be chosen based on the type of analysis intended. For example, in terms of very basic statistics, usually at least three replicates per category are required. This will allow for basic diversity statistics, the identification of discriminant taxa and a core microbiome (e.g., [43]). However, for more advanced ecological modelling to be applied, usually a minimum of five to six replicates are required; this will usually satisfy criteria for the null models which often underpin community assembly analysis (e.g., [18,44,45]). Finally, for even more involved methods such as network inferences, upwards of 35 samples per category are often necessary to obtain reliable results (e.g., [46,47]). 

When planning a microbiome study, it is often advisable to plan backwards—thinking about what types of statistical analyses are best going to help answer the experimental questions. Although it may seem counterintuitive, there is often more power and impact in designing studies that test fewer conditions but include more replicates. In this way we can go beyond basic diversity estimates and identify and pinpoint specific ecological patterns from the data. 

## 3. Current Trends in Data Processing

Several processing pipelines have been developed which include a series of steps and programs employed to align, denoise, and remove spurious sequences: e.g., MOTHUR [48], QIIME 2 [49], and KRAKEN2 [50]. While these pipelines are flexibly designed and updated for quality assurance, users generally follow a series of steps without significant deviation. Such pipelines typically resolve species through an operational taxonomic unit (OTU) approach (often clustered at 97% similarity), or an amplicon sequencing variant (ASV) approach [51]. While ASVs have gained popularity in recent years, ongoing research has shown that they often yield similar diversity trends as OTUs [52], and that such an approach can artificially split bacterial genomes into clusters [53]. Conversely, it has recently been proposed that an ASV-based approach better represent the sequence diversity of functional genes, where it can be difficult to identify an appropriate threshold [54]. 

**Figure 1:**
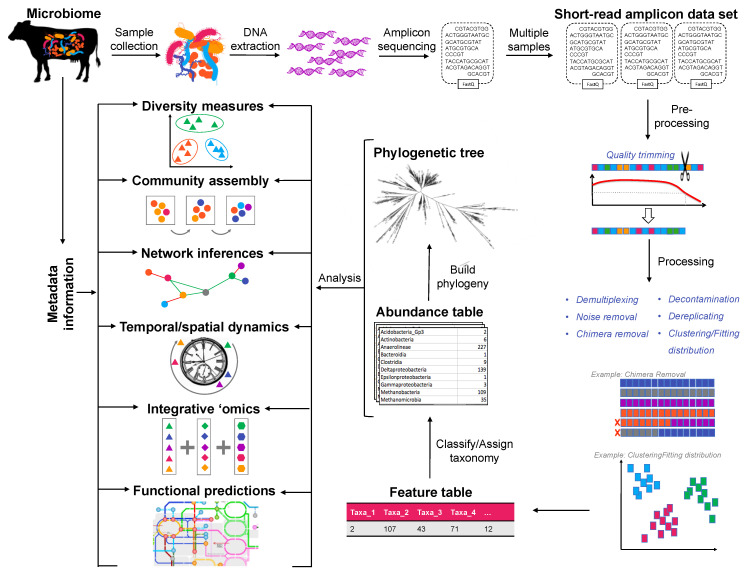
Summary of amplicon sequencing workflow. Basic diversity analysis are only a small fraction of available types of analyses available for microbiome analysis.

Within processing pipelines, tools such as VSEARCH [55] or USEARCH [56] were traditionally used for denoising, dereplicating and clustering into OTUs. The current release of USEARCH (v.11) includes a denoising option which will generate ZOTUs (zero-radius OTU). These are considered suitable for diversity analysis, although where traditional OTUs at 97% similarity may include more than one species, one species may have more than one ZOTU [57,58]. Conversely, DADA2 [59] and Deblur [60] are useful for resolving ASVs. Deblur, in particular uses error profiles to obtain putative error-free sequences from sequencing data with better sensitivity and specificity than other available tools [60]. The product of these processing steps is to produce a feature table (Figure 1), wherein ASVs or OTUs are grouped, but taxonomy has yet to be assigned. 

In either an ASV- or OTU-based approach, taxonomy is resolved by aligning the sequences against a reference database up to species level, where possible. The alignment process is most often implemented as a classifier (Figure 1), such as the traditional naïve Bayesian classifier (NBC). Recently however, the Bayesian lowest common ancestor (BLCA) algorithm has been proposed to give shades-of-grey assignments (with confidences) by considering the phylogeny of the reference ‘hits’ [61]. This approach, as opposed to NBC, is able to provide greater taxonomic resolution. 

Several taxonomic databases are available, including Greengenes [62], RDP [63], AutoTax [64] and SILVA [22]. Currently, SILVA ribosomal RNA gene database (release 138) is the most comprehensive (containing 436,680 sequences) for 16S rRNA data. However, species-level taxonomy is not curated within this database with species classifications often falling into the category of ‘uncultured’ or ‘metagenome’. In contrast, environment-specific and highly curated databases have also been developed, such as MiDAS (specific to wastewater treatment microbiomes) [24], TaxAss (for freshwater microbiomes) [25] or RefSoil (for soil microbiomes) [65], which help facilitate enhanced species-level assignments. Once taxonomy has been assigned and classified (yielding the final abundance table), a phylogenetic tree can be constructed. Both the abundance table and phylogenetic tree are required for downstream statistical analysis. 

## 4. Traditional Measures of Diversity: Revisiting the Basics

The most fundamental ecological questions center around biodiversity, which is vital in terms of ecosystem function. Recently, for example, the idea of whether or not diversity begets diversity was explored. The authors concluded that for low-diversity systems, diversity does promote more diversity, but that eventually ecosystem diversity will reach a plateau [66]. Indeed, substantial efforts have been made in both macro- and microbiology to quantify and monitor diversity patterns. This is typically accomplished using alpha and beta diversity measures, by highlighting taxa that are dominant, differential, or persistent, and analyzing them within the context of similarity or dissimilarity between samples. These analyses are often performed in R using *vegan* [67] and the *phyloseq* package [68]. 

Alpha diversity is one of the most common and well-established metrics for measuring diversity. It is a means of calculating, at a local scale, the richness (number of observed taxa) evenness (distribution of abundances of the observed taxa), or both [69]. There are multiple approaches to calculating the alpha diversity of a sample, but the three most widely-accepted methods are the Shannon entropy [70], the rarefied richness [71], and/or the Simpson index [72]. Rarefied richness (rarefied to the lowest number of sequencing reads) is a useful means of comparing the number of observed taxa within a sample, without giving any consideration to abundances. It is often employed to track how the overall quantity of species changes over time, space or experimental conditions. Alternatively, Shannon entropy is a measure of the balance of taxa within a sample in terms of abundances. A high value indicates that all taxa within the community are equally abundant, while reduced values generally suggest that a sub-group of taxa are becoming more dominant within the community [70]. Additionally, a unified family of diversity indices called Hill Numbers exist. These are typically alpha diversity-generating formulas (^q^D), which are often parameterized with q, for example, q = 0 gives richness estimate, q = 1 gives Shannon entropy, and q = 2 gives inverse Simpson index. Depending on the value of q used, the returned diversity can result in more or less emphasis on rare/abundant species [73]. Notably, there are several biases and assumptions associated with the estimation of alpha diversity and the application of measurement error models has been suggested to be useful in adjusting for any uncertainty [74]. 

While alpha diversity is the diversity within a sample, beta diversity compares diversity between samples. There are three common beta diversity distance metrics: (i) Bray–Curtis; (ii) UniFrac; and (iii) the weighted UniFrac [75,76,77]. Bray–Curtis distances are calculated based on abundances and work very well for ecological datasets [78]. UniFrac, however, is based on phylogenetic distances (thus requiring a phylogenetic tree) and considers how closely related the taxa within a community are by looking for the unique fraction of the phylogenetic tree. Notably, it is based on presence/absence data and therefore emphasizes rare community members [75]. Weighted UniFrac, however takes both counts and phylogeny into account and therefore is biased toward the dominant groups [76]. The beta diversity for any given distance metric is generally visualized on either a principal coordinate analysis plot (PCoA), or a non-metric multi-dimensional scaling (NMDS) plot [79]. PCA-type methods rely on eigen value/eigen vector decomposition, often of the covariance matrix. This is accomplished by generating a new abundance table, which has the same number of dimensions as the original table, but all the variability is shifted to the first dimension. They typically preserve distances between the samples in a reduced representation, but at the expense of loss of variability. NMDS, on the other hand, transforms the data (by optimizing a stress function) to however many dimensions the data is to be visualized for. Whilst it captures better variability, it comes at the cost of conserving distances in the reduced space, mainly maintaining their monotonic relationships. In general, PCoA plots are more accurate than NMDS for studies with fewer sample numbers and many features (ASVs/OTUs) [80]. In all cases, however, we look for clustering patterns between the samples, where similar communities will cluster together. Finally, a simple tool allows us to assess if any of the samples diverge too much from the average beta diversity of the ecosystem. This technique is called the local contribution to beta diversity (LCBD) [81], is available in R as a part of the *adespatial* package [82], and can enable us to have a very simple, quantifiable measure for “microbial dysbiosis”.

The next most common types of diversity analysis include: (i) highlighting the abundant fraction of the microbiome, usually with a heatmap or a taxa-bar plot; and (ii) looking for taxa that are differential (changing between categories); (iii) persistent (core); or (iv) conditionally rare and abundant (CRAT). Differential taxa, or discriminant taxa can be calculated in several ways using a variety of thresholds [17,83]. Notably, sPLS-DA analysis, available through the *mixOmics* package (discussed later in Section 8) uses an advanced algorithm to identify discriminant taxa within microbiomes [84]. Core microbiome analysis is equally versatile in terms of how a core microbiome is defined. Notably, the core microbiome has previously been defined as any feature present in at least 85% of the samples [85]. Furthermore, several detection thresholds can be applied in terms of abundance to sort the core microbiome from the low-abundant core, to the high-abundant core yielding a 2-dimensional representation of the taxa that persist within the system. This model has now been applied to several ecosystems including wastewater treatment facilities [44], chicken [17], and fish microbiomes [83]. Finally, an emerging way to look at both the abundant and rare fractions of the microbiome is to focus on those taxa whose abundance is conditional, CRAT taxa [86]. 

These few analyses constitute the main bulk of data analytical techniques typically used in published manuscripts. However, as ‘omics modalities are becoming cheaper, there is a world-over shift to incorporate additional modalities (flow-cytometry, transcriptomics, metabolomics, proteomics) to fill in the gaps that arise with static nature of 16S rRNA datasets as some species may be active but in low abundance or vise versa [43,87,88]. These demand a new way of consolidating all the information by developing methodologies that not only give correlations between the datasets, but also have a discrimination component which reduce the features to set of absolute minimal features that capture the main patterns. 

Additionally, we often also want to capture underlying ecological principles particularly in terms of the environmental habitat where these microbes are observed and to assign them specific roles, i.e., whether they are generalists of specialists; whether they are influenced by the environment; whether they share a niche with other microbes; whether they have symbiotic relationships or compete for resources; or whether they are predominately active (throughout the spatial or temporal gradients) or are transient and proliferate sporadically in response to a biotic or abiotic influence. These explorations require bespoke analytical techniques, the majority of which have been explored in macro-ecology literature and are slowly being adapted to microbiome research. 

## 5. Identifying Mechanisms Driving Microbial Community Assembly

Despite decades of research, development, and process optimization, several outstanding questions remain regarding the ecology of engineered systems. Paramount among these is how dynamic communities assemble both during system start-up and throughout all subsequent operational phases. If we can determine whether assembly mechanisms are predictable, and pinpoint precisely when and how we can manipulate these processes, we will be one step closer to being able to manage and control these communities to serve particular functions. Key to this is understanding the driving forces shaping the microbiome [89].

Ecologists have been working on identifying distributions of species patterns among sites, trying to come up with simple quantifiable metrics on incidence tables (presence/absence) such as *Coherence* (degree to which spatial patterns could be collapsed into a single dimension); *Species Turnover* (which describes the number of species replacements after the collapse); and *Boundary Clumping* (how the edges of species are distributed across the dimensions) [90]. These three measures, adopted to microbiome research [91] are then useful to understand the metacommunity structure. Relatedly, an additional framework describes species distributions along environmental gradients, manifesting themselves as metacommunity structures with the following discernable patterns: *Random* (no gradients or patterns in species found); *Checkerboards* (species pairs have mutually exclusive distrbutions and such pairs occur independently); *Nested* (nested subsets are observed); *Evenly spaced Gradients* (species ranges are arranged more evenly); *Gleasonian* (gradients results in species turnover, but species ranges are random); and *Clementsian* (discrete communities that replace each other as a group) [92]. Whilst these are useful methods, they do not take into account species’ abundance, nor phylogenetic relatedness—a limitation that has resulted in minimal uptake. 

While we still lack a general theory to explain how communities are assembled, community ecologists have, over time, converged towards a framework which acknowledges that these processes are either stochastic or deterministic. Deterministic influences can either be biotic or abiotic which leave their influence on the observed community structure, whether on the composition, or on the phylogeny. “Stochasticity”, on the other hand, refers to completely random effects shaping the microbiome [93]. Elucidating these community assembly processes, if deterministic, then provides a means to look for patterns in the surrounding environment that are responsible (e.g., some physico-chemical characteristics). This is highly relevant for experimental designs where the goal is to alter the community structure to make it optimal in view of certain performance criteria (for example, optimal functioning of wastewater treatment, or reducing microbial dysbiosis in pathological conditions). 

To this end, new models, usually requiring only sequencing abundance data (such as an ASV- or OTU-table) and sometimes a phylogenetic tree, are continuously being developed to help identify and quantify ecological mechanisms driving change in these communities. Most often, these are based on a null modelling procedure, where community structure (composition or phylogeny) is perturbed by creating in situ artificial variations (typically 999), by conserving a certain property of the original samples (typically, alpha diversity). Depending on the choice of the mathematical metric used, its deviation from the original community structure to the average of when applied to altered structures, has the power to reveal ecological phenomena. 

Notably, Quantitative Process Estimates (QPE) uses null modelling to quantify assembly within an ecological framework based on selection and dispersal mechanisms [94]. Specifically, it classifies assembly mechanisms as (i) *variable selection*, when selective environmental conditions result in high compositional turnover; (ii) *homogenous selection*, when static environmental conditions result in consistent selective pressure; (iii) *dispersal limitation*, when low rates of dispersal (movement of microorganisms in space) result in high community turnover (this drives ecological drift); (iv) *homogenizing dispersal*, when high compositional turnover is fueled by high dispersal rates; or (v) “*undominated*”, when compositional turnover is neither the result of selection nor dispersal [18,95]. QPE holds several advantages over other types of assembly quantification tools: (i) it incorporates both phylogenetically informed, and phylogenetically uninformed data making it better suited for understanding ecological phenomena—whereas several other available methods do not take phylogeny into account (such as the stochasticity ratio (SR), discussed next); (ii) with QPE we can measure the relative contribution of different processes acting simultaneously upon a community; and (iii) QPE allows for the identification and comparison of dominant assembly mechanisms under different conditions. A recent extension to this framework is by incorporating phylogenetic bin-based null model analysis which is essentially the same procedure but at finer bin-levels with sufficient phylogenetic signal [96].

An alternative to divulging stochastic and deterministic assembly mechanisms is through the normalized stochasticity ratio (NST) approach [97]. This method, while useful, is not nearly as informative as QPE, especially in terms of exact mechanisms. For each community, it yields a percentage value called the stochasticity ratio (SR), indicating the quantity of stochastic processes that shaped that community—implying that all other remaining processes were deterministic. It is, however, a straightforward way of tracking the contribution of stochasticity. 

Quantifying and assessing changes in biological diversity are central aspects of many ecological studies, yet accurate methods of estimating biological diversity from microbial samples remains a challenging problem. Although Hill numbers were first proposed several decades ago as alpha diversity-generating formulas, their extension and parameterization to beta diversity space is only recent, providing a means of identifying ecological assembly mechanisms [98], particularly to see if the differences between samples are driven by stochastic or deterministic processes. Additionally, it has been suggested that Hill numbers can also be used to examine the relationships between different community members by incorporating phylogenetic information. This could provide additional information about functional differences between communities [98]. 

Another common framework for discussing community assembly mechanisms is in terms of niche vs. neutral processes [99,100]. Although sometimes used interchangeably, these concepts are not equivalent to deterministic and stochastic processes. While neutrality reflects the ecological equivalence of species (when demographic rates such as birth, death and dispersal are identical), stochasticity suggests random, probabilistic, variation in species’ demographic rates. Likewise, niche processes suggest differences between species’ mean demographic rates, while determinism implies an absence of random, probabilistic, changes in species’ demographic rates [101]. Using simulated stochastic and deterministic metacommunities, Tucker et al. (2016) were able to use the abundance-based ß-null deviation measures to differentiate between niche and neutral community assembly. This has since been applied to understand the community assembly of fish (Atlantic cod) microbiomes [45] and marine bioplankton [19]. 

An elegant, and easily applied tool for examining microbial ecosystems is the competitive lottery model for clade-based assembly [29]. The model assumes that phylogenetically related organisms are functionally similar, sharing similar gene content, preferential niche space and metabolisms—all giving rise to intense clade-level competition. Within a given clade/group, ‘winners’ would arise, being more ‘fit’ to the given conditions, or simply having arrived to the community and established first (priority effects). These ‘winner’ ASVs make up a majority of the abundance within their clade—capturing >90% of the groups abundance [29]. Application of this model to anaerobic digestion (AD) systems helped to describe not only how overall abundances shifted, but identified specific ‘winners’ which either adapted to the given environment, becoming ‘winners’, or were unsuited and lost their ‘winner-status’ [18,44]. The power of the lottery model is that it identifies specific ‘winning’ ASVs for each clade/group, which can be tracked across conditions, allowing for a deeper understanding of how different trophic groups are behaving, responding and functioning. 

Continuing to think about how niche spaces influence microbial communities, recent work has advanced our ability to use microbiome sequence data to identify *generalists* and *specialists* within complex communities [102,103]. While generalists are taxa that inhabit a broad range of environments, or environmental gradients, specialists occupy a narrow and discriminatory niche space. Using the publicly available package in R, *MicroNiche*, we can use abundance tables and corresponding data to differentiate between generalist and specialist species, but also to identify species that are positively or negatively correlated with environmental data of interest [103]. Similarly, we can consider the specificity of microorganisms to a given environmental gradient. Using the R package *Specificity*, the quantity of specificity can be calculated and correlated across different environmental covariates. Additionally, the package will identify taxa that are specific to a given environmental covariate [31]. 

## 6. Network Inferences: Identifying Relationships and Revealing Complexity

At the very basic level, co-occurrence networks are a useful way to interpret microbial community dynamics. These networks can handle the scale and diversity of microbiome data, and have the added advantages of being able to identify microbial interactions/associations [104], identify network re-organizations under varying conditions [105], identify ‘hub’ species [106] or estimate diversity [107]. Recently, networks were even used to predict a ‘die score’—identifying organisms within the community that were most likely to be eliminated under various conditions [108]. While compositional approaches to network modelling are varied and continue to evolve [109], here we will highlight three different approaches, beyond simple correlation-based approaches. 

First, SPIEC-EASI (**SP**arse **I**nvers**E C**ovariance Estimation for **E**cological **AS**sociation **I**nference) remains a robust and popular choice for network inference [110] which is derived from Graphical Lasso method, a sparse penalized maximum likelihood estimator for the concentration or precision when the species abundances are modelled as a Gaussian distribution. SPIEC-EASI was developed specifically to address two issues: (i) that amplicon-based studies yield ASV abundances that are compositional (abundances are relative to one another) and (ii) that results of such studies are often biased by poor sampling depth. It yields network visualizations (at a specified taxonomic rank), that indicate relative abundances, network clustering within the community and positive and negative associations between groups. It is a flexible tool for evaluating the adaptation, development and interactions within a microbial community. Recently, Boolean logic has been applied to microbial communities, allowing abundance patterns to emerge in new ways, via Boolean multi-dimensional arrangements [104]. The idea revolves around compartmentalizing interactions between two or more species by finding thresholds at which the species in an interacting ecosystem can be deemed as present or absent to infer *co-presence*, *one-way*, and *co-exclusion* relationships by calculating the probability scores on the inferred compartments. Moreover, these various relationships can be identified in multiple dimensions, where 2D relationships are between two interacting species, and 3D relationships are identified between three interacting species. 

Any one change in community dynamics can cause a cascade of additional disturbances within the system. In many cases understanding how to maintain stability would be beneficial [111,112]. The complexity-stability paradigm predicts that stable ecosystems have an upper limit to their overall complexity [113]. Therefore, it would be unlikely to observe communities comprising both a large number of species and a high degree of interspecific interactions. Instead, to maintain stability, microbial communities have evolved to be either small and highly interacting, or large but weakly interacting. This trade-off between community complexity and stability emphasizes the role of interspecific interactions in governing overall species richness of a community [47]. Generally, calculating the complexity-stability across different communities has been challenging because while the species richness can, in theory, be measured, the degree of interspecific interactions is more challenging.

However, identifying relationships of interest between microbial species is useful to reveal symbiosis and antagonism, as microbial species rarely act alone, striving for resources with a cascade of pathways where multiple species enable conversion of substrates. These relationships can be associations as well as causal relationships, and May’s stability criteria [113] models interactions between species as a Generalised Lotka Volterra Model. Herein one can use the interaction matrix $A_{ij}$ (effect of species j on species i) to reveal the stability-complexity relationship of the ecosystem which can be derived as a curve that satisfies $\alpha \sqrt{nC}<1$ where $\alpha^{2}$ and C are the variance and density (“*connectance*”) of the non-zero off-diagnoal elements of $A_{ij}$. However, this all depends on accurate network inference, limited by concise framework to incorporate ecological understanding of interacting species.

Recently, Yonatan et al. (2022) has addressed the May’s stability criteria by calculating “effective connectance” (degree of interactions) without the need to infer the networks explicitly. This is done by analysing the beta diversity measures such as ‘dissimilarity’, and “overlap” for N(N-1)/2 sample pairs for given N samples in a category. The authors suggest that in addition to evaluating complexity-stability relationships, the effective connectance may give insights into the degree to which local perturbations, which cause shifts in the abundance of one or a few species, will propagate and affect the whole community. Communities with high effective connectance will be more substantially affected by a few changes in abundance than communities with low effective connectance [47]. 

## 7. Over Space and Time: Measuring Temporal/Spatial Dynamics

Space and time are arguably two of the most important variables in many microbiome studies. Sampling communities over time or space allows for the assessment of community development [18,44], microbial evolution [114], biogeography [115], global distributions [116], dispersal rates [95], functional robustness [30], and/or responses to treatments or perturbations [117]. Such studies benefit from analytical methods which evaluate (i) trends over time according to variables of interest; (ii) magnitude of change within individual categories; and/or (iii) the inherent variation in complex biological systems [118]. Many analytical options are continuously developed; here we highlight some interesting/useful options. 

For assessing beta diversity on temporal or spatial scales, zeta diversity has been recently proposed. This can better detect shifts and transitions, and emphasizes the roles of rare versus abundant species in the microbial consortia [119,120]. Next, built directly into QIIME 2, *q2-longitudinal* is a software plugin which offers multiple methods for the streamlined analysis and visualization of longitudinal data, providing valuable information on temporal trends [118]. Several functions are available including analysis of (i) volatility; (ii) feature-volatility; (iii) linear mixed effects (LME); (iv) first differences and (v) first distances, among several others. Microbial volatility is best described as the variance in microbial abundance, diversity or other metric over time. Changes in volatility can be indicative of system disturbances and thus can be a useful parameter in the context of engineered bioprocesses. The most common way to examine change over time is to compare the average relative abundances of taxa over time, usually via a box plot or a heat map. Unfortunately, such approaches usually work by targeting the most abundant microorganisms, ignoring features that are actually associated with specific time points. Using the feature-volatility function, machine learning regressors (random forests by default) learn the structure of the data and identify all features that are predictive of different categories (time points). Next, LME is able to test whether a taxa’s relative abundances are impacted by time, or other available parameters. Finally, the first differences and first distances actions allow for the assessment of the rate of change between time points [118]. 

Another interesting tool is the phylogenetic recruitment model, which examines microbial species succession over temporal scales [121]. The method works by detecting the order in which new species are detected and is linked to phylogenetic diversity (PD) estimates. The authors use the dispersion parameter (D), which is calculated based on the probability of detection of new species by fitting a logistic error model on temporal changes in phylogenetic diversity (PD) estimates. The value of D determines the primary recruitment mechanisms. If D = 0, then all species have an equiprobable chance of recruitment (neutral). If D > 0, then phylogenetically divergent taxa (to the taxa detected in the previous time-points) are preferentially added to the community (overdispersed). In contrast, if D < 0, then phylogenetically similar taxa are preferentially added to the community (underdispersed—or *nepotistic*). This model has been implemented to understand recruitment trends in microbiomes [45,121] where the communities were generally nepotistic and recruitment was disrupted in ‘perturbed’ communities. 

Recently, an R package called *splinectomeR* was introduced, allowing the assessment of statistical changes within a longitudinal dataset using three key functions: *permuspliner*, *sliding_spliner* and *trendyspliner* [122]. These functions can test hypotheses regarding observations over time without transforming or collapsing the data points, as is done in many existing longitudinal studies. The first function, permuspliner, tests overall variability and noise between two groups in longitudinal studies. With this we can identify taxa that change longitudinally/spatially. The second function, sliding_spliner, gains information on the specific time when change occurred, identifying periods/spatial ranges when there is change in taxa abundances between multiple categories. Importantly, this function requires a large dataset (50–100 data points). Finally, the third function, trendyspliner, tests for a significant non-zero overall trends in a single population over time, identifying whether an abundance profile is linear or non-linear. *splinectomeR* achieved these statistical analyses using summary splines and randomly permuted distribution to assess the significance of the observed magnitude of change between groups or trends over time. 

An alternative approach has made advances in integrating temporal datasets of different types. If, for example, a timeseries of major taxa is observed, then on temporal basis we can find a subset of these taxa that cluster together under the ‘time-omics’ framework [123]. This is especially useful when a study contains both microbial community data and other types of heterogenous biological, environmental, metabolome, chemical or phenotypic data—which are reduced to subset of series, significant in the context of temporal dynamics of microbiome experiments. The method selects key temporal features with strong associations within an “automated” clustering driven by principal coordinate analysis (PCA), where each dimension comprises of two clusters. This way, for a given category, species are clustered that have similar temporal evolution (using Silhouette metric). Each species is modeled as a function of time, considering all the variabilities of the different replicates in a linear-mixed model spline framework. Moreover, the fitted splines enable us to predict/interpolate time points that might be missing. One can then use sparse PCA to retain the most important features (taxa). The method is not only useful for microbiome data, but can also be used for any other modality (flowcytometry, metabolomics, etc.) where there is temporal data acquisition, the only difference being the normalization procedure. Finally, if a study includes matching longitudinal data (i.e., multi-modalities: metagenomics + metabolomics), then we can also get clusters with features across multiple datasets—useful in giving a mechanistic understanding. 

One of the most compelling questions for microbial ecologists is, whether, or to what degree a microbial community is deterministically dependent on its initial/previous composition. To address this question, a new model MTV-LMM (Microbial Temporal Variability Linear Mixed Model) has been proposed [124]. MTV-LMM is a linear mixed model that can be applied to predict the temporal dynamics of microbial communities in longitudinal studies. Application of this model will identify time-dependent taxa—those affected by the past composition of the microbiome—these microbes can then be used to describe the temporal trajectories of the microbiome. Moreover, MTV-LMM introduces a concept termed ‘time-explainability’, a measure of the fraction of temporal-variance that can be explained by the community profile at previous time points. The application MTV-LMM to engineered systems, for example, would yield valuable information on time-dependent compositional patterns allowing for the prediction and modulation of these microbial communities. 

Other notable efforts have been made to give a more mechanistic understanding of species richness across different spatial scales [125]. Species richness is one of the most common measures of biodiversity, but the specific ecological processes driving change are difficult to disentangle. To this end, a framework was developed wherein variation in species richness can be decomposed into three components: (i) species abundance distribution; (ii) species density; and (iii) species spatial aggregation/distribution. By constructing several types of rarefaction curves, the relative contribution of the three components of species richness can be measured across scales. These methods are available in the R package *mobr*. Such tools will help ecologists move beyond single-scale analyses such as species richness alone. 

## 8. Integrative ‘Omics: Combining Multiple Datasets

With advances in computational biology we are now in a position to go beyond simply observing microbial communities, and can rather identify meaningful connections by integrating several ‘omics technologies (targeted sequencing strategies, proteomics and/or metabolomics) through sophisticated dimensionality reduction algorithms [126]. These not only optimize for correlation between multiple datasets but also give the discriminatory power to filter out features that do not change in a case–control relationship (multiple-category comparisons). While simply merging data sets might lead to false positive hypotheses, three types of integrative approaches have been developed: (i) data complexity reductions; and (ii) supervised or (iii) unsupervised integration. Several packages have been developed to computationally handle multivariate analysis of such large biological data sets. 

Recently, *STATegra* was designed as a conceptual framework, designed to be as user-friendly as possible for the identification of biological features and pathways within large data sets [127]. It is available in R through the *STATegRa* Bioconductor package. There are four primary steps with various tools available at each. In the first instance, each ‘omic data set is analysed separately (univariate analysis). Following this, the data sets are jointly analysed for component analysis, feature identification and exploratory analysis.

Notably, *mixOmics* is a user-friendly R package which takes a systems-biology approach, focusing on probing relationships through data exploration, dimension reduction, integration and visualization [84]. It uses multivariate projection-based methodologies in order to handle large data sets and highlight variation in biological systems; and their relaxed assumptions about data distribution make the methods highly flexible, fitting a wide range of study designs. Importantly, the methods allow for the identification of discriminant groups within biological data sets, using several approaches. Firstly, the sparse projection to latent structure discriminant analysis (sPLS-DA) can be applied as a supervised analysis of one data set, yielding discriminant features at a given taxonomic level. Additionally, when a study consists of several independent data sets measured on the same predictors, MINT can be used to integrate these studies and identify common discriminants. Both sPLS-DA and MINT can be easily applied to 16S rRNA data sets [17,83,128]. Finally, DIABLO (Data Integration Analysis for Biomarker discovery using Latent variable approaches for Omics studies) allows for the integration of data sets using the same biological samples measured on different ‘omics platforms (i.e., metagenomics and proteomics) [84,129]. Notably, a common bottleneck to experimental design is that parallel measurements from various ‘omics technologies are required. However, the ongoing development of such integrative frameworks will continue to be essential to the discovery of new biology as data sets become increasingly complex.

## 9. Meta-Data Integration: Using Regressions to Identify Key Taxa

Typically studies in microbial ecology use a case–control, multi-factorial design, seeking out spatial or temporal relationships between microbiome data and other sources of variation from the environment. Often, the aim of these controlled experiments is to manipulate the environment to modulate the microbiome. Sources of variation can take the form of continuous or categorical variables including clinical, socio-economic, chemical, or other parameters. Identifying relationships between these covariates and the microbiome allows us to understand and predict how complex communities respond to stress, environmental perturbations, or other disturbances. This is usually achieved using various types of regression analysis [130].

Recent years have seen a growing interest in the development of new methods for multivariate analysis of microbiome data [33,131]. In particular, joint species distribution models, which use random effects to identify correlations between environmental variables and predictions of species abundances, have garnered increased interest [33,132]. Such models assume that species will respond jointly to the environment as well as to each other, and thus have the potential to pinpoint the causes of species co-occurrence patterns and identify microorganisms that are positively or negatively associated with a given co-variate [132,133]. A key approach to this class of statistical modeling is the generalized linear latent variable model (GLLVM) [134], which is capable of handling datasets containing thousands of species—a common challenge when the number of observed species greatly outnumbers the number of samples [135,136]. Previously, the application of GLLVMs has been computationally slow and impractical for large datasets. However, *gllvm*, a new package for R, is able to quickly apply GLLVMs to large multivariate datasets, with features allowing for model selection and high-quality visualizations [33,136]. 

There are several challenges to the statistical analysis of microbiome data. As mentioned previously, the size and multidimensional nature of such data are a significant challenge. Additionally, the compositional nature of microbiome data presents another significant challenge, i.e., the fact that changes in the abundance of one species induce changes in the observed abundances of the other species (that abundances are relative to one another). Compositional data analysis (CoDA) is an alternative framework which seeks to provide methods to appropriately handle complex compositional data [137]. While there are many CoDA approaches, a subset are formulated from generalized linear models, each having specific constraints [130,138]. Among these are *selbal,* which relies on a selection of microbial balances [139]; clr-lasso, which is a simple penalized regression [140,141,142] on centered log-ratio (clr)-transformed data [138]; and *coda*-*lasso,* which performs penalized regression on a log-contrast regression model [32]. In particular, *coda*-*lasso* works particularly well when the aim is the identification of taxa most associated with a given environmental variable [138]. Notably it is now available within *coda4microbiome*, a new R package for the analysis of microbiome data within the CoDA framework [32]. 

## 10. Predictive Functional Modelling: Using Structure to Infer Function

The advantage of functional profiling over taxonomic profiling is that it assesses what a microbial community can do, rather than simply who is present [143,144]. Advances in sequencing technologies have been accompanied by progress in the comprehensiveness of the associated databases. Better databases means that the predictive modelling which can be accomplished using targeted gene sequencing (usually the 16S rRNA gene as a cheap alternative to shotgun metagenomics [145]) now offers somewhat better exposition on putative functional behavior. This is especially true when combined with new tools that are able to greatly reduce or eliminate amplification biases and/or errors based on amplicon-genome linkages [146]. 

PICRUSt2 [147] is a prime example of an accessible and much-improved database. It boasts ~20,000 genomes while is predecessor, PICRUSt1, only included 2011 genomes. Using the QIIME 2 plugin KEGG enzymes and MetaCyc pathway predictions can be found. Such predictions are highly dependent on the number of pathways available for the reference genomes. The algorithm consistently predicts pathways that yield greater than 0.8 correlation with the actual pathways observed using shotgun metagenomic equivalents as highlighted by the authors [147]. While Picrust2 has been shown to be more accurate in its predictive capacity, care should still be taken during interpretation as they are still only putative estimates. 

Similarly, Tax4Fun2 [148], FAPROTAX [149] and BugBase [150] have all been developed to predict function from taxonomic profiles. *Tax4Fun2* is an R package that makes such predictions based on 16S rRNA gene sequence data regardless of sequencing platform. The algorithm yields a list of KEGG Orthologs (KOs) relating to specific functions and is additionally able to calculate functional gene redundancies [148]. FAPROTAX is a manually constructed database that maps prokaryotic taxa to function. It converts abundance profiles into putative functional group abundance profiles. Notably, an individual taxon may be affiliated with multiple functions within FAPROTAX [149]. BugBase is an algorithm which predicts organism-level coverage of functional pathways but also provides biologically interpretable phenotypes such as oxygen tolerance, biofilm formation and pathogenic potential. Notably, BugBase can also be customized to identify particular traits of interest [150]. All four approaches provide putative functional mapping when used with 16S rRNA sequence data. 

Recently, there have also been advances in how to correctly estimate beta diversity between functions. Traditionally, Bray–Curtis distances (or any other count measure) were applied on functional abundance (KEGG Orthologs: KOs) tables obtained from PICRUSt2 or any other metabolic prediction software. These mathematical measures assume each feature (typically microbes) to be independent, which does not hold true for functional enzymes. This is mainly because there is redundancy in KOs spaces with multiple KOs serving the same function, some available to some species, and others to a different set of species. Therefore, to capture the hierarchy and inherent dependences between KOs, a new beta diversity measure called Hierarchical Meta-Storms (HMS) [151] has been introduced. This takes into account the information of which KOs are implicated in the definition of pathways (as a hierarchical structure), and then starts at the bottom level of these hierarchies and then propagates the abundances upwards to give a weighted dissimilarity measure. This then provides higher sensitivity for detecting variations in upper-level metabolic pathways between samples.

Finally, a simple approach to assessing microbiome stability measures functional robustness by linking changes in the structure of the community to specific functional shifts [30]. Robustness is an important factor of the microbial community, especially for engineered ecosystems, which often depend on community resilience for process stability [152]. While functional dynamics are best examined via metagenomic or metatranscriptomic datasets, the Taxa Function Robustness measures the degree to which a shift in a community’s structural (taxonomic) profile (using amplicons) will result in a change in its functional capacity, particularly two parameters, “*Attenuation*” and “*Buffering*” summarizes the functional robustness of communities in each samples, that can reveal not only overall functional robustness, but can also help look at specific pathways of interest. It is based on the underlying assumption that a community’s functional capacity is directly related to the genes available within that community [30]. When the structural profile shifts, certain genes (and therefore functions) may be lost entirely, while others may remain—redundant within other, more stable species. With this tool we can track changes in functional capacity across time, according to environmental conditions, or between different systems. While all of these methods for predicting functionality of microbiomes are useful and highly informative, it is worth reiterating that they only give putative functionalities and should therefore be interpreted with caution. Indeed, they maybe be highly useful for generating hypotheses rather than drawing strong conclusions. 

## 11. Conclusions

Microbial ecology is not just about simply describing which microbes increase or decrease in abundance based on study design. Advances in informatics have provided at our fingertips the ability to identify “unknown” factors that come into play in assembling microbial communities; reveal spatial and temporal patterns; and give a snapshot of how stable or complex a system is, and whether the microbial community is able to withstand environmental perturbations. Simply relying on genomic based identification and proliferation of microbial species is not sufficient to infer mechanistic patterns. For this, we need to incorporate other modalities, and meta-data, including physico-chemical parameters, which is only recently possible with the advancement in integrated ‘omics techniques. Shotgun metagenomics remains very popular by virtue of recovering microbial genomes and can give metabolic potential of microbial species, however, suffers from accurately estimating the diversity, by virtue of depth of resolution. Therefore, studies that focus on ecology and taxonomic expanse of the microbial communities are best suited to use amplicon-based surveys, which now are well advanced in terms of databases. Here, we have highlighted methods that are in routine use and serve as a guideline to perform microbial community analyses. 

## Data Availability

Not applicable.
